# Perfluorocarbon liquid-assisted vitreo-dissection in eyes with firmly adherent posterior hyaloid

**DOI:** 10.1186/s12886-022-02715-1

**Published:** 2022-12-07

**Authors:** Ihab Abdel Aziz, Moaaz M. Hussein, Yousef A. Fouad

**Affiliations:** 1 Al Mashreq Eye Center, Cairo, Egypt; 2 The Memorial Institute for Ophthalmic Research, Cairo, Egypt; 3 Department of Ophthalmology, Electricity Hospital, Cairo, Egypt; 4 Department of Ophthalmology, Ain Shams University Hospitals, Cairo, Egypt

**Keywords:** Pars Plana Vitrectomy, Perfluorocarbon liquid, PFCL, Posterior vitreous detachment, Vitreo-dissection

## Abstract

**Background:**

Induction of posterior vitreous detachment (PVD) is a critical step during pars plana vitrectomy. Multiple techniques and utilities have been proposed for assistance with this step with no consensus on the safest and most effective means, especially in eyes with firmly adherent posterior hyaloid. Viscodissection or the utilization of perfluorocarbon liquid (PFCL) can be used to dissect the posterior hyaloid and widely adherent epiretinal membranes.

**Methods:**

A technique of PFCL dissection of the posterior hyaloid in eyes with abnormal adhesion of the posterior hyaloid. After core vitrectomy, breaking into the posterior hyaloid face is made via active aspiration and cutting or a sharp dissection. This is followed by active and slow injection of PFCL into the potential space between the posterior cortical vitreous and the neurosensory retina. A wave of PFCL propagates anteriorly causing “vitreo-dissection” of the peripheral cortical vitreous.

**Results:**

The technique was effective and safe in 8 successive cases, 4 cases with vitreoretinal traction syndrome and 4 with diabetic tractional membranes.

**Conclusion:**

The technique can be considered in cases with abnormal firmly adherent posterior hyaloid when induction of PVD proves difficult.

**Supplementary Information:**

The online version contains supplementary material available at 10.1186/s12886-022-02715-1.

## Background

Induction of posterior vitreous detachment (PVD) is one of the critical steps of pars plana vitrectomy (PPV) for the treatment of multiple retinal disorders [1]. Those include preretinal tractional membranes seen with proliferative diabetic retinopathy [2], and anomalous vitreomacular interface that causes traction (vitreomacular traction, VMT) [3]. The degree of preoperative PVD has been recently shown to correlate with visual outcomes and reoperation rate for tractional retinal detachment (TRD) in eyes with PDR that underwent PPV for non-clearing vitreous hemorrhage [4].

Classical teaching of how induction of PVD should be performed encompasses active aspiration of the cortical vitreous over the optic disc margin using the vitreous cutter. However, some cases may have a firmly adherent posterior hyaloid, and newer smaller-gauge probes have more distal openings on the probe’s shaft that reduces their grasping power, both factors could potentially lead to a more difficult PVD induction [5]. In such cases, forced induction of PVD may be associated with a higher incidence of iatrogenic breaks, which can lead to a poorer surgical outcome [6].

Multiple techniques and instruments have been proposed to induce a break in the posterior hyaloid and facilitate PVD. Those include using a diamond duster membrane scraper (DDMS) [7], a sharp instrument [5], or a membrane scraper made by polypropylene loop [8]. In 2019, Babu et al. [9] proposed the “mega Weiss-ring” for induction of PVD in eyes with retinal detachment. In this technique, Perfluorocarbon Liquid (PFCL) is injected after core vitrectomy, followed by gentle stroking of the posterior hyaloid using a DDMS to create a defect that is circumferentially enlarged to form a 4–5 disc diameter ring with rolled-out edges. Lifting the edges using an internal limiting membrane peeling forceps allows for the PFCL to fill the potential space between the posterior hyaloid and retina, and peripherally extends the PVD into completion.

Here, we provide a modification on the mega Weiss-ring technique to induce and propagate PVD assisted by PFCL in eyes with firmly adherent posterior hyaloid.

## Methods

**Fig. 1:**
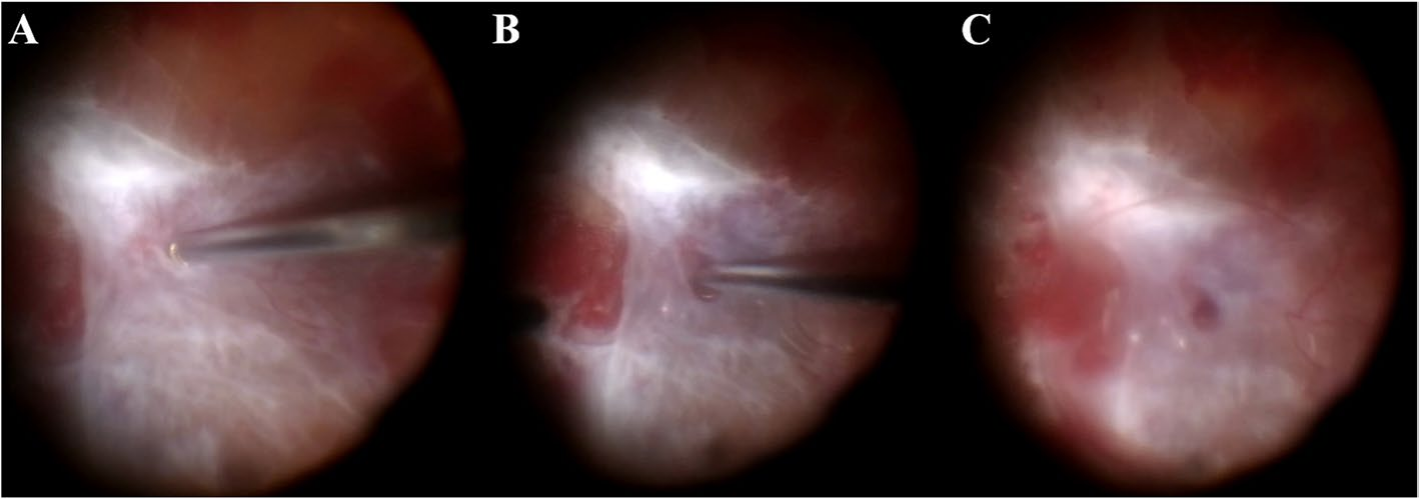
Vitreo-dissection technique using PFCL. Initial creation of a hole within the posterior hyaloid using active aspiration and cutting by the vitrectomy probe (**A**), followed by introduction of the PFCL cannula into the hole directed towards the potential space between the posterior cortical vitreous and the neurosensory retina (**B**), and injection of PFCL to form a bubble that propagates, elevating the posterior cortical vitreous away from the retina (**C**)

This technique was successfully used with both 23- and 25-guage PPV. After adequate core vitrectomy, staining of the posterior cortical vitreous is done by injection of 0.1 to 0.3 mL of triamcinolone acetate suspension. Multiple trials of PVD induction are initially made by active aspiration of the cortical vitreous over the optic disc (Fig. 1A). If this fails, a sharp instrument (28-gauge needle with bent tip) is used to penetrate the posterior hyaloid combined with active aspiration as described by Ellabban et al. [5].

Injection of PFCL is then carried out through the hole in the posterior hyaloid, in the potential space between the posterior cortical vitreous and neurosensory retina in a manner that mimics the hydro-dissection or visco-dissection during cataract surgery in which the lenticular cortex is dissected from the inner capsular surface (Fig. 1B). However, controlled slow injection is crucial from a safety standpoint to avoid iatrogenic break formation; this can be carried out using an automated injection method via infusion attachment to the PFCL cannula and foot pedal control or manual injection, with the opening of the cannula aimed away from the fovea in both cases. A test outside the eye is important to ensure cannula patency and modify injection speed prior to injection inside the eye. A successful injection is heralded by a slow wave of PFCL propagating centrifugally, and separating the posterior hyaloid face from the inner paracentral then peripheral retinal surface (Fig. 1C). Continuation of the conventional steps of vitrectomy could then be carried out (supplemental digital content 1).

In our experience, this technique was most useful in cases with abnormal, firmly adherent posterior hyaloid. We utilized this technique with excellent results in eyes that underwent PPV for VMT or diabetic TRD. Possible complications are rare and included failure to propagate the PVD, the potential iatrogenic injury to the retina by the PFCL cannula which could lead to retinal break or bleeding, or the creation of iatrogenic breaks during dissection by the PFCL.

## Results and discussion

We describe a simple and relatively safe technique for induction and propagation of PVD in eyes with abnormal adhesion of the posterior hyaloid. We have successfully performed this technique in 4 eyes with VMT and 4 eyes with diabetic TRD, with no detectable complications. The technique allows for quick propagation of a PFCL wave that separates the posterior cortical vitreous from the retinal surface; thus, avoiding the complications seen with sharp dissection [5].

Recently, a “mega Weiss-ring” technique was described by Babu et al. [9] for eyes with rhegmatogenous retinal detachment. In that technique, PFCL is injected prior to creation of a hole in the posterior hyaloid and then let to passively “slide” into the space between the cortical vitreous and retina by lifting the edges of the posterior hyaloid. In our technique, active rather than passive introduction of the PFCL allows for a more rapid dissection of the posterior vitreous face. The “mega Weiss-ring” technique also depends on the ability of PFCL to flatten the neurosensory retina against the retinal pigment epithelium, making it unsuitable for cases with advanced preretinal proliferation, traumatic and long-standing detachments. Our active “vitreo-dissection” obviates such contraindications.

Earlier on, Arevalo had described the “en bloc perfluorodissection” for TRD [10]. The technique also utilizes PFCL injection into a hole created in the mid-peripheral posterior hyaloid in eyes with advanced diabetic retinopathy and epimacular membranes. This allows for the complete separation of epiretinal tissue in an “en bloc” manner and was proven safe in the series of eyes operated on by the author.

Some limitations exist in our report, mainly the small sample size since we consider this a “proof-of-concept” study, and the grouping of different pathologies (PDR and VMT) together. An ongoing effort aims to assess outcomes of the technique in a larger cohort with specific pathologies. PFCL toxicity is another limitation that may hinder the use of our described technique, especially in cases where PFCL use had not been originally indicated for assisting the surgical repair. Short-term injection of PFCL in the vitreous cavity has been demonstrated to be well-tolerated in humans. Direct retinal toxicity is often related to subretinal retention of the liquid [11]. In the cases where we utilized the technique, no pre-existing breaks or macular holes were detectable preoperatively, potentially obviating the occurrence of such complication. The only possible route for PFCL to reach the subretinal space would be the creation of an iatrogenic break during the initial steps of PVD induction, which we did not observe in any of our cases. Nevertheless, such a scenario cannot be ruled out and should warrant caution during utilization of this technique.

## Supplementary Information

**Additional file 1: Supplementary Digital Content 1.** Video demonstration of the “vitreo-dissection” technique. Gentle injection of PFCL into the potential space between the posterior cortical vitreous and the neurosensory retina.

## Data Availability

All data generated or analyzed during this study are included in this published article.

## Abbreviations

DDMS: diamond duster membrane scraper
PFCL: perfluorocarbon liquid
PPV: pars plana vitrectomy
PVD: posterior vitreous detachment
TRD: tractional retinal detachment
VMT: vitreomacular traction
