# Supplementary material for: Perfluorocarbon liquid-assisted vitreo-dissection in eyes with firmly adherent posterior hyaloid
Source: BMC Ophthalmol. 2022 Dec 7;22:475. doi: 10.1186/s12886-022-02715-1 (PMC9727894; doi:10.1186/s12886-022-02715-1)
Supplement: Supplementary file 1 — Additional file 1: Supplementary Digital Content 1. Video demonstration of the “vitreo-dissection” technique. Gentle injection of PFCL into the potential space between the posterior cortical vitreous and the neurosensory retina. [file 12886_2022_2715_MOESM1_ESM.zip › Supplementary Digital Content Title and Legend.docx]

**Supplementary Digital Content 1**

**Title:** Video demonstration of the “vitreo-dissection” technique

**Legend:** Gentle injection of PFCL into the potential space between the posterior cortical vitreous and the neurosensory retina.
